# Plasma Ceramides and Sphingomyelins and Sudden Cardiac Death in the Cardiovascular Health Study

**DOI:** 10.1001/jamanetworkopen.2023.43854

**Published:** 2023-11-17

**Authors:** Lee B. Bockus, Paul N. Jensen, Amanda M. Fretts, Andrew N. Hoofnagle, Barbara McKnight, Colleen M. Sitlani, David S. Siscovick, Irena B. King, Bruce M. Psaty, Nona Sotoodehnia, Rozenn N. Lemaitre

**Affiliations:** 1 Department of Medicine, University of Washington, Seattle; 2 Department of Epidemiology, University of Washington, Seattle; 3 Departments of Laboratory Medicine and Pathology, University of Washington, Seattle; 4 Department of Biostatistics, University of Washington, Seattle; 5 New York Academy of Medicine, New York; 6 Department of Internal Medicine, University of New Mexico, Albuquerque; 7 Department of Health Systems and Population Health, University of Washington, Seattle

## Abstract

**Importance:**

Sphingolipids, including ceramides and sphingomyelins, may influence the pathophysiology and risk of sudden cardiac death (SCD) through multiple biological activities. Whether the length of the fatty acid acylated to plasma sphingolipid species is associated with SCD risk is not known.

**Objective:**

To determine whether the saturated fatty acid length of plasma ceramides and sphingomyelins influences the association with SCD risk.

**Design, Setting, and Participants:**

In this cohort study, multivariable Cox proportional hazards regression models were used to examine the association of sphingolipid species with SCD risk. The study population included 4612 participants in the Cardiovascular Health Study followed up prospectively for a median of 10.2 (IQR, 5.5-11.6) years. Baseline data were collected from January 1992 to December 1995 during annual examinations. Data were analyzed from February 11, 2020, to September 9, 2023.

**Exposures:**

Eight plasma sphingolipid species (4 ceramides and 4 sphingomyelins) with saturated fatty acids of 16, 20, 22, and 24 carbons.

**Main Outcome and Measure:**

Association of plasma ceramides and sphingomyelins with saturated fatty acids of different lengths with SCD risk.

**Results:**

Among the 4612 CHS participants included in the analysis (mean [SD] age, 77 [5] years; 2724 [59.1%] women; 6 [0.1%] American Indian; 4 [0.1%] Asian; 718 [15.6%] Black; 3869 [83.9%] White, and 15 [0.3%] Other), 215 SCD cases were identified. In adjusted Cox proportional hazards regression analyses, plasma ceramides and sphingomyelins with palmitic acid (Cer-16 and SM-16) were associated with higher SCD risk per higher SD of log sphingolipid levels (hazard ratio [HR] for Cer-16, 1.34 [95% CI, 1.12-1.59]; HR for SM-16, 1.37 [95% CI, 1.12-1.67]). Associations did not differ by baseline age, sex, race, or body mass index. No significant association of SCD with sphingolipids with very-long-chain saturated fatty acids was observed after correction for multiple testing (HR for ceramide with arachidic acid, 1.06 [95% CI, 0.90-1.24]; HR for ceramide with behenic acid, 0.92 [95% CI, 0.77-1.10]; HR for ceramide with lignoceric acid, 0.92 [95% CI, 0.77-1.09]; HR for sphingomyelin with arachidic acid, 0.83 [95% CI, 0.71-0.98]; HR for sphingomyelin with behenic acid, 0.84 [95% CI, 0.70-1.00]; HR for sphingomyelin with lignoceric acid, 0.86 [95% CI, 0.72-1.03]).

**Conclusions and Relevance:**

The findings of this large, population-based cohort study of SCD identified that higher plasma levels of Cer-16 and SM-16 were associated with higher risk of SCD. Future studies are needed to examine the underlying mechanism of these associations.

## Key Points

**Question:**

Do associations of sphingolipids with sudden cardiac death (SCD) vary based on the length of the acylated saturated fatty acid?

**Findings:**

In this cohort study of 4612 participants aged 65 years or older with 215 SCD events, plasma ceramides and sphingomyelins with palmitic acid were associated with 34% and 37% higher SCD risk, respectively, per higher SD of log plasma levels.

**Meaning:**

These findings suggest that sphingolipids with palmitic acid are associated with increased risk of SCD, whereas those containing very-long-chain saturated fatty acids are not.

## Teaser

This cohort study addresses whether ceramide and sphingomyelin species containing very-long-chain saturated fatty acids were associated with a reduced risk of sudden cardiac death (SCD) and whether species containing palmitic acid were associated with an increased risk of SCD among participants in the Cardiovascular Health Study.

## Introduction

Sudden cardiac death (SCD) is a leading cause of mortality, accounting for 180 000 to 300 000 deaths in the US annually.^1,2,3^ Ventricular fibrillation is the most common rhythm at the time of SCD, and cardiovascular disease (CVD) is the most common underlying pathophysiology.^2,4,5,6,7^ A number of risk factors have been associated with SCD, including CVD and its risk factors as well as atrial fibrillation and electrophysiological variables. Understanding the biochemical mechanisms underlying SCD will allow for better treatment and prevention measures.

Sphingolipids have been reported to be important risk factors for CVD, including heart failure and atrial fibrillation, 2 conditions that markedly elevate cardiac arrest risk.^8,9,10,11,12^ Ceramides and sphingomyelins are in the family of sphingolipids. They are purported to be integral to a multitude of cardiovascular-related signaling pathways^13,14^ and play a role in oxidative stress, inflammation, fibrosis, and remodeling.^8,15,16^ Interestingly, the length of the saturated fatty acid acylated to the sphingolipid species may influence its biological activity.^17^ Lemaitre et al^18,19^ previously reported associations of sphingolipid species with heart failure and diabetes, both conditions associated with SCD, with differential associations depending on saturated fatty acid length.

Whether circulating levels of ceramides and sphingomyelins are associated with SCD and whether the associations vary with the length of the saturated fatty acid are not known. This study was hypothesis driven, based on previous work showing associations of erythrocyte membrane very-long-chain saturated fatty acids (VLSFAs) with a lower risk of SCD^20^; associations of ceramides and sphingomyelins with palmitic acid (Cer-16 and SM-16) with a higher risk of total mortality; and associations of VLSFA-acylated ceramides and sphingomyelins with lower total mortality.^21^ The goals of this analysis were to address the hypothesis that ceramide and sphingomyelin species containing a VLSFA are associated with a reduced risk of SCD and species containing palmitic acid are associated with an increased risk of SCD within the Cardiovascular Health Study (CHS).

## Methods

### Study Design and Setting

The CHS is a prospective cohort study of CVD risk factors among community-dwelling adults 65 years or older. The study design and procedures are described in detail elsewhere.^22,23^ In 1989 and 1990, 5201 participants were selected at random from lists of Medicare beneficiaries and recruited from 4 field centers located in Pittsburgh, Pennsylvania; Washington County, Maryland; Forsyth County, North Carolina; and Sacramento County, California. An additional cohort of 687 predominantly Black individuals was recruited in 1992 to 1993 with the same methods from the same counties except Washington County. The CHS was approved by the institutional review board at each field center, and all study participants gave written informed consent. This study followed the Strengthening the Reporting of Observational Studies in Epidemiology (STROBE) reporting guideline.

### Data Collection

Between June 10, 1989, and July 15, 1999, participants underwent annual study examinations that included personal interviews, physical examinations, laboratory assessments, and diagnostic tests. Collected data included height, weight, blood pressure, questions about tobacco and alcohol use, medical history, and an electrocardiogram. At the 1992 to 1993 examination, levels of the following analytes were measured: high-density lipoprotein (HDL) and low-density lipoprotein (LDL) cholesterol, C-reactive protein (CRP), fibrinogen, N-terminal pro–brain-type natriuretic peptide (NT-proBNP), and troponin T. Sphingolipid plasma levels were measured at the 1992-1993 or 1994-1995 study examination, and patient characteristics from that examination formed the study baseline for the analysis.

Participant race was self-reported in the CHS. Race data were collected for the present study because we wanted to determine whether the study results applied to different racial and ethnic groups.

### Sphingolipid Measurement

Fasting EDTA plasma samples collected at the 1994-1995 examination (n = 4026) and at the 1992-1993 examination for participants without a 1994-1995 plasma sample (n = 586) were used for ceramide and sphingomyelin assays. Plasma samples were stored at −70 °C until they were extracted. Extracted sphingolipids were quantified using liquid chromatography–tandem mass spectrometry as described in detail previously.^18^ We examined 8 sphingolipid species: ceramide and sphingomyelin with acylated palmitic acid (Cer-16 and SM-16, respectively), arachidic acid (Cer-20 and SM-20, respectively), behenic acid (Cer-22 and SM-22, respectively), or lignoceric acid (Cer-24 and SM-24, respectively); Cer-24 comprised the summed concentrations of 2 ceramide species with distinct sphingoid backbones. In determining sphingolipid concentrations, a pooled EDTA plasma sample was used as a single-point calibrator and added to each batch in 5 replicates. An independent pool of EDTA plasma served to provide a quality control sample that was added to each batch in duplicate; coefficients of variation for each of the sphingolipid measurements were less than 20% over the whole period of laboratory assays, which included 52 batches.

### Identification of Outcomes

Adjudication of SCD in the CHS was performed as previously published.^24,25^ In brief, information was gathered on cardiovascular events and deaths from hospital medical records, interviews with next of kin and primary health care clinicians, death certificates, and autopsy and coroner’s reports where available. Cause of death and nonfatal myocardial infarctions (MIs) were adjudicated by the CHS Event Committee. All adjudicated cardiac deaths were reviewed and adjudicated for SCD by a cardiologist (N.S.) using the following case definition: a pulseless condition in a previously stable individual presumed due to ventricular arrhythmia without evidence of either a noncardiac cause of death or progression of previous hypotension or heart failure. All events occurred outside the hospital or in the emergency department. By definition, cases could not have a life-threatening noncardiac comorbidity or be under hospice or nursing home care. A blinded second physician review was used, with high interreviewer agreement of 88%.^24^ Used for comparison analysis, nonfatal MIs were adjudicated by physician committee as previously described.^24,26^

### Statistical Analysis

Data were analyzed from February 11, 2020, to September 9, 2023. There were no exclusions made for the SCD analyses. Associations of sphingolipid levels with SCD were assessed using Cox proportional hazards regression models, and statistical significance was determined with the Wald score. Participants began accruing time after their sphingolipid measurement and were followed up until an SCD event, death, dropout, or November 30, 2012. Sphingolipid levels were log_2_ transformed, and results are presented per SD difference in log concentration of each sphingolipid. We assessed 3 primary sets of models with a priori selected baseline characteristics as adjustment terms: model 1 (the minimally adjusted model) included adjustment for baseline age, sex, race, and study site; model 2 (the adjusted model) included model 1 additionally adjusted for body mass index (BMI), treated hypertension, HDL and LDL cholesterol levels, smoking, and prevalent type 2 diabetes and CHD; and model 3 (the primary model) included model 2 additionally adjusted for one of the other species: the Cer-16 and SM-16 models included adjustment for Cer-22 and SM-22, respectively; the Cer-20, Cer-22, and Cer-24 models included adjustment for Cer-16; and the SM-20, SM-22, and SM-24 models included adjustment for SM-16.

Missing values of HDL (n = 203) and LDL (n = 280) cholesterol levels were multiply imputed using information on age, sex, race, BMI, and smoking. Twenty imputed data sets were generated, and model-fitting results were pooled using standard methods.^27^

In sensitivity analyses, we repeated our primary analysis with additional adjustment for prevalence of heart failure, atrial fibrillation, chronic kidney disease by estimated glomerular filtration rate (eGFR), and chronic obstructive pulmonary disease (model 4). Using values measured at the 1992-1993 examination in additional models, we adjusted for heart rate, QT interval, and QRS interval (model 5); and log-transformed CRP, NT-proBNP, and troponin T levels (model 6).

For the nonfatal MI analyses, patients with a history of MI were excluded, leaving 4092 patients for the analysis. Associations of sphingolipid levels with nonfatal MI were performed using the same Cox proportional hazards regression models as with SCD. The same adjustment terms were used, and the data are presented in a comparable fashion.

We report 95% CIs for the hazard ratios (HRs). However, to correct for multiple comparisons, we assessed statistical significance at a threshold of 2-sided *P* < .0063 (0.05/8 sphingolipid species) by Bonferroni correction. The assumption of proportional hazards was not violated in any analysis, and there was no evidence of departure from linearity. Analyses were performed using Stata, version 16 (StataCorp LLC).

## Results

**Figure 1.**
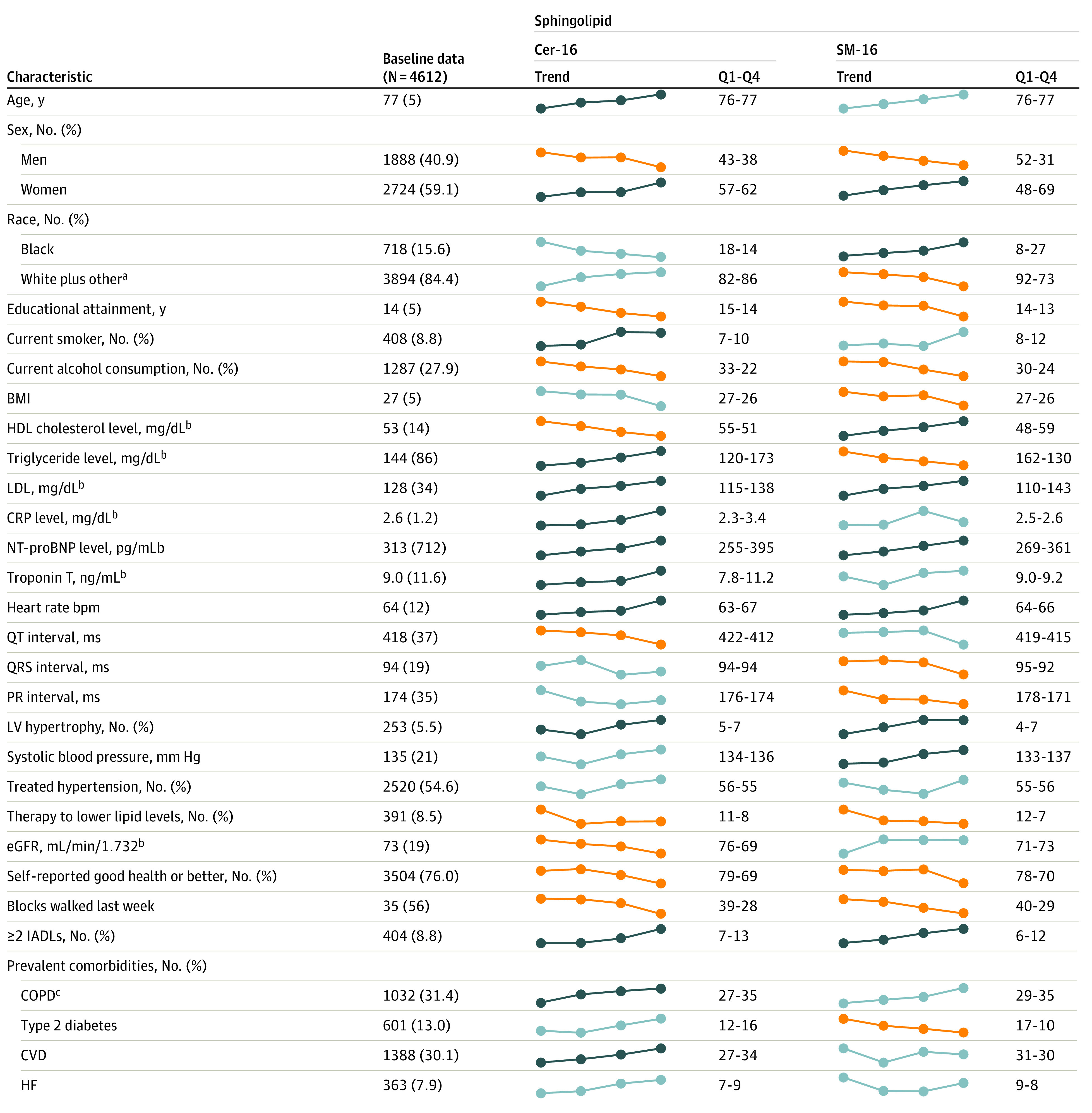
Baseline Characteristics and Trends Across Quartiles of Sphingolipid Levels Among Cardiovascular Health Study Participants Unless otherwise indicated, data are expressed as mean (SD). Unadjusted linear and logistic regression models were used to assess significant (*P* < .0018; 0.05/28 characteristics) associations of log-transformed sphingolipids with each characteristic. The dotted lines represent means or percentages of each characteristic across quartiles of each of the sphingolipids; dark blue lines indicate significant positive trends, orange lines indicate statistically significant negative trends, and light blue lines indicate *P* > .0018. Q1 indicates the mean or percentage of each characteristic among participants with a sphingolipid level in the lowest 25% of the distribution; Q4 indicates the mean or percentage among participants with a sphingolipid level in the highest 25% of the distribution. BMI indicates body mass index (calculated as weight in kilograms divided by height in meters squared); Cer-16, ceramide with palmitic acid; COPD, chronic obstructive pulmonary disease; CRP, C-reactive protein; CVD, cardiovascular disease; eGFR, estimated glomerular filtration rate; HDL, high-density lipoprotein; HF, heart failure; IADLs, instrumental activities of daily living; LDL, low-density lipoprotein; LV, left ventricle; NT-proBNP, N-terminal pro–brain-type natriuretic peptide; Q1, the lowest quartile of the distribution; Q4, highest quartile of the distribution; SM-16, sphingomyelin with palmitic acid. To convert CRP to mg/L, multiply by 10; HDL and LDL cholesterol to mmol/L, multiply by 0.0259; NT-proBNP to ng/L, multiply by 1.0; triglycerides to mmol/L, multiply by 0.0113; troponin T to μg/L, multiply by 1.0. ^a^Other includes American Indian or Alaska Native, Asian, and other. ^b^Measured in 1992 to 1993 for all participants. ^c^Information available for 3282 participants.

The baseline characteristics of the 4612 included participants and their distributions across quartiles of each of the sphingolipids are presented in Figure 1 and eTable 1 in Supplement 1. The CHS participants examined were older adults (mean [SD] age, 77 [5] years) and predominantly female (2724 women [59.1%] vs 1888 men [40.9%]). A total of 6 participants (0.1%) were American Indian; 4 (0.1%) were Asian; 718 (15.6%) were Black, 3869 (83.9%) were White, and 15 (0.3%) were other races or ethnicities. Smoking and alcohol consumption were uncommon, and most participants were free of underlying prevalent CVD at baseline (Figure 1). Higher concentrations of LDL cholesterol were associated with higher plasma levels of all 8 sphingolipid species in univariate analysis, whereas association with age, sex, race, and other clinical factors differed by sphingolipid species type (Figure 1 and eTable 1 in Supplement 1).

**Figure 2.**
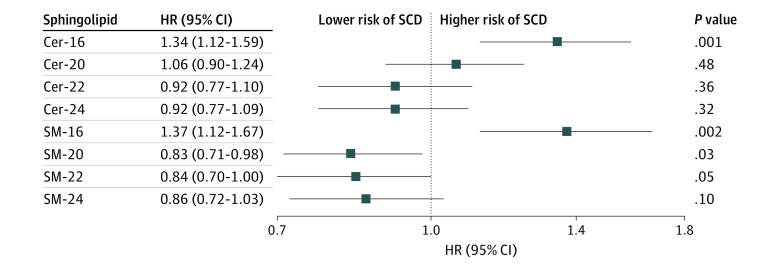
Risk of Sudden Cardiac Death (SCD) per Higher SD of Log Sphingolipid Levels Based on 215 Events Among 4612 Patients Hazard ratios (HRs) and 95% CIs were adjusted for age, sex, race, study site, body mass index, treated hypertension, high- and low-density lipoprotein cholesterol levels, smoking, and prevalent type 2 diabetes and coronary heart disease. Additionally, ceramide and sphingomyelin with palmitic acid (Cer-16 and SM-16, respectively) include adjustment for ceramide with behenic acid (Cer-22) and sphingomyelin with behenic acid (SM-22), respectively; ceramide with arachidic acid (Cer-20), behenic acid (Cer-22), and lignoceric acid (Cer-24) include adjustment for Cer-16; and sphingomyelin with arachidic acid (SM-20), behenic acid (SM-22), and lignoceric acid (SM-24) include adjustment for SM-16.

Over the median follow-up of 10.2 (IQR, 5.5-11.6) years, 215 SCD cases were identified (incidence rate, 5.5/1000 person-years). Each SD log difference in plasma Cer-16 was associated with a 34% higher risk of SCD (HR, 1.34 [95% CI, 1.12-1.59]) and in plasma SM-16, with a 37% higher risk of SCD (HR 1.37 [95% CI, 1.12-1.67]) in models adjusted for age, sex, race, study site, BMI, HDL and LDL cholesterol levels, smoking, treated hypertension, prevalent type 2 diabetes, CHD, and levels of other sphingolipids (Figure 2).

Higher levels of ceramides containing a VLSFA were not associated with a difference in SCD risk (HR for Cer-20, 1.06 [95% CI, 0.90-1.24]; HR for Cer-22, 0.92 [95% CI, 0.77-1.10]; HR for Cer-24, 0.92 [95% CI, 0.77-1.09]). Each SD log difference of sphingomyelins with a VLSFA showed a nonsignificant lower risk of SCD (HR for SM-20, 0.83 [95% CI, 0.71-0.98]; HR for SM-22, 0.84 [95% CI, 0.70-1.00]; HR for SM-24, 0.86 [95% CI, 0.72-1.03]) (Figure 2).

To further explore the mechanism of the association of sphingolipids with SCD, we performed sensitivity analyses with additional adjustment for prevalent clinical disease (chronic obstructive pulmonary disease, heart failure, atrial fibrillation, and chronic kidney disease by eGFR) or for electrocardiographic risk factors for SCD (heart rate, QT interval, and QRS interval). These only minimally attenuated the associations of Cer-16 and SM-16 with SCD (eTable 2 in Supplement 1, models 4 and 5). A model that included adjustment for CRP, troponin T, and NT-proBNP levels showed an attenuated association (eTable 2 in Supplement 1, model 6).

**Figure 3.**
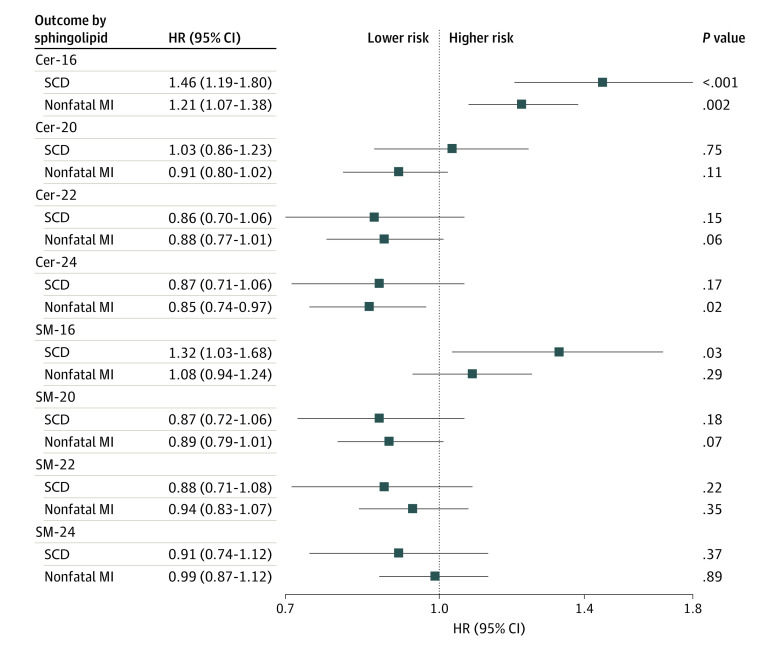
Risk of Sudden Cardiac Death (SCD) and Incident Nonfatal Myocardial Infarction (MI) per Higher SD of Log Sphingolipid Levels Participants with a history of MI were excluded from both analyses. Of the remaining 4092 participants, there were 160 instances of SCD and 412 instances of nonfatal MI. Hazard ratios (HRs) and 95% CIs are presented, adjusted for age, sex, race, study site, body mass index, treated hypertension, high- and low-density lipoprotein cholesterol levels, smoking, and prevalent type 2 diabetes and coronary heart disease. Additionally, ceramide and sphingomyelin with palmitic acid (Cer-16 and SM-16, respectively) include adjustment for ceramide with behenic acid (Cer-22) and sphingomyelin with behenic acid (SM-22), respectively; ceramide with arachidic acid (Cer-20), behenic acid (Cer-22), and lignoceric acid (Cer-24) include adjustment for Cer-16; and sphingomyelin with arachidic acid (SM-20), behenic acid (SM-22), and lignoceric acid (SM-24) include adjustment for SM-16.

We next examined the association with nonfatal MI to determine whether the associations with SCD were specific to SCD (Figure 3). In a subset of patients without prior MI (n = 4092), each SD log difference of Cer-16 was associated with a 21% higher risk of incident MI (n = 412; HR, 1.21 [95% CI, 1.07-1.38]) and a 46% higher risk of SCD (n = 160; HR, 1.46 [95% CI, 1.19-1.80]). The SD log difference of SM-16 was not associated with nonfatal MI but was associated with a 32% higher risk of SCD (HR, 1.32 [95% CI, 1.03-1.68]).

Given the moderate correlation of 0.45 between Cer-16 and SM-16 (eFigure in Supplement 1), we ran models including both sphingolipids in the same model and found attenuation of both sphingolipids’ associations with SCD (eTable 3 in Supplement 1), suggesting that both lipid species partly account for the observed SCD risk association. In interaction analysis, there was no evidence that associations of the sphingolipids with SCD were significantly modified by age, sex, race, or BMI (eTable 4 in Supplement 1).

## Discussion

In this cohort study of older adults followed up prospectively for SCD, plasma levels of Cer-16 and SM-16 were associated with a higher risk of SCD. These associations were independent of other risk factors and did not differ by subgroups, including age, sex, race, and BMI.

Ceramides and sphingomyelins are membrane, intracellular, and circulating lipids in the sphingolipid family of bioactive lipids. Ceramides are composed of a fatty acid acylated to a sphingoid base, with sphingomyelins having the addition of a choline head group.^28^ Sphingolipids are synthesized de novo within most cells, and blood levels of sphingomyelins and ceramides may be a systemic metabolic signature.^28,29,30^ Ceramides as well as sphingomyelins play a role in oxidative stress, inflammation, fibrosis, and remodeling,^8,15,16^ all of which may influence the risk of SCD.

Sphingolipid species containing saturated fatty acids of different lengths show different biological activities in both experimental^17^ and patient association studies.^31^ Diabetes and heart failure are important risk factors for SCD, and previous investigations have found associations of ceramides and sphingomyelins with these outcomes,^18,19^ as well as atrial fibrillation^32^ and mortality,^21^ based on the length of the acylated fatty acid. Another group found an association with cardiovascular mortality as well.^33^ Previous investigations^34,35,36,37^ have also reported associations of plasma phospholipid VLSFAs with lower risk of these conditions. Importantly, Lemaitre et al^20^ have reported that higher levels of several VLSFAs (arachidic, behenic, and lignoceric acids) measured in erythrocyte phospholipids, including phosphoglycerolipid and sphingomyelin fatty acids, are associated with a lower risk of SCD.

Our hypothesis is that ceramide and sphingomyelin species with acylated palmitic acid can increase the risk of SCD in 2 compounding ways: a direct effect on cardiac electrophysiology and an indirect effect through ceramide release and a resultant accentuated inflammatory response. First, efficient electrical signaling requires concerted activation of multiple ion channels, and altered electrical coupling is a hallmark of cardiac arrhythmia. In the case of left ventricular tachyarrhythmias that are the cause of SCD, these are generally propagated via cell-to-cell electrical signaling through gap junctions, highly regulated areas of electrical signaling. Lipid rafts, which are enriched in sphingomyelins, play an important role in this process, both by their influence on ion channels^38,39^ and by proper assembly of these microdomains.^40,41^ Aberrant electrical signaling, possibly due to altered electrophysiological cellular communication, can predispose to SCD.

Second, the observed parallel associations of Cer-16 and SM-16 may exist because membrane-bound sphingomyelins can release ceramide via the enzymatic action of neutral sphingomyelinase,^14^ with sphingomyelins providing spatial control and sphingomyelinase providing temporal control of ceramide release. This enzyme is reported to be rapidly activated in ischemia and ischemic-reperfusion injury,^42^ representing a potential stress response mechanism for ceramide release in the setting of MI.^43^ The resulting ceramide release is associated with multiple detrimental pathways, including inflammation and apoptosis,^8,16,44,45^ potentially increasing both the damage caused by ischemia^45,46^ and the risk of SCD. Interestingly, while Cer-16 was associated with a higher risk of both nonfatal MI and SCD (though with a greater magnitude of association with SCD), SM-16 was associated only with SCD and not with nonfatal MI. Although there may be some mechanistic overlap between SCD and nonfatal MI, the potential effect of SM-16 may be more specific to SCD, considering that membrane localization of sphingomyelin may be more relevant to the aberrant electrical signaling of SCD. Interestingly, the association of Cer-16 with both MI and SCD remained after adjustment for LDL and HDL cholesterol levels, triglyceride levels, diabetes prevalence, eGFR, and race, suggesting that these associations may be mechanistically independent of these factors. Associations of ceramides and sphingomyelins with SCD were attenuated after adjustment for CRP, NT-proBNP, and troponin T levels, suggesting a possible connection to inflammatory or myocardial wall stress-related mechanisms.

Of note, the sphingolipids in the present study were associated with LDL cholesterol levels, consistent with their presence in apolipoprotein B–containing complexes.^47^ Future studies could help elucidate sphingolipids associated with different lipoproteins.

The primary prevention strategy for SCD is placement of implantable cardioverter defibrillators in high-risk individuals. It has been suggested that sphingolipids be included in risk assessment of coronary artery disease and cardiovascular events,^48,49,50,51,52^ and the present work supports their further exploration. Future studies in independent populations should evaluate whether risk prediction is improved by the inclusion of sphingolipid risk species.

### Strengths and Limitations

The strengths of this study include its prospective design, the detailed and thorough assessment of ceramide and sphingomyelin species and multiple risk factors that might affect SCD risk, and the number of identified cases with SCD. The study is a first step in showing the associations of specific species of plasma sphingolipids with risk of SCD, a difficult-to-study phenotype.

This study also has some limitations. First, because it was observational in nature, we cannot infer causation; future studies, including mendelian randomization techniques and animal studies, are needed to address causality. Second, the study participants were of African and European descent, with a mean baseline age of 77 years, thus our findings may not be generalizable to individuals of other races or ethnicities or to younger populations. Third, given that the study was based on a single measurement, we were unable to distinguish confounding from mediation. Fourth, there might be variations in an individual’s sphingolipid profile over time, for example with diet,^53^ and circulating levels were reported to increase with age over decades.^54^ Any measurement error introduced by individual variation over time would likely be random and bias the study toward the null hypothesis. Finally, levels of HDL and LDL cholesterol, CRP, NT-proBNP, and troponin T were measured only at the 1992-1993 study examination, and their values may not accurately reflect their levels at baseline in participants whose sphingolipid levels were measured in the 1994-1995 examination.

## Conclusions

The findings of this cohort study of participants from the CHS, suggest novel associations of ceramide and sphingomyelin species with SCD. These associations may help refine risk stratification for SCD among populations being considered for cardioverter defibrillator implantation. If the associations prove to be causal, reducing the levels of ceramide and sphingomyelin species with acylated palmitic acid may be a useful therapeutic target for the prevention of SCD. Additional studies will be needed to establish determinants of plasma levels of ceramide and sphingomyelin species and to evaluate whether ceramide and sphingomyelin levels may have clinical utility as potential components of SCD risk scores.

## Notes

Supplement 1. **eTable 1.** Mean Baseline Characteristics and Trends Across Quartiles of Sphingolipids Among 4612 CHS (Cardiovascular Health Study) Participants **eTable 2.** Sensitivity Analyses for Risk of Sudden Cardiac Death per SD Higher Log Sphingolipid Level Based on 215 Events Among 4612 Participants **eTable 3.** Mutual Adjustment of Species With Palmitic Acid, Cer-16 and SM-16, in Addition to Model 3 Adjustment **eTable 4.** Interactions With Age, Sex, Race, and BMI (Body Mass Index) **eFigure.** Correlation Matrix Among the Sphingolipid Species of Interest

Supplement 2.
Data Sharing Statement
