# Supplementary material for: Plasma Ceramides and Sphingomyelins and Sudden Cardiac Death in the Cardiovascular Health Study
Source: JAMA Netw Open. 2023 Nov 17;6(11):e2343854. doi: 10.1001/jamanetworkopen.2023.43854 (PMC10656644; doi:10.1001/jamanetworkopen.2023.43854)
Supplement: Supplement 1. — eTable 1. Mean Baseline Characteristics and Trends Across Quartiles of Sphingolipids Among 4612 CHS (Cardiovascular Health Study) Participants eTable 2. Sensitivity Analyses for Risk of Sudden Cardiac Death per SD Higher Log Sphingolipid Level Based on 215 Events Among 4612 Participants eTable 3. Mutual Adjustment of Species With Palmitic Acid, Cer-16 and SM-16, in Addition to Model 3 Adjustment eTable 4. Interactions With Age, Sex, Race, and BMI (Body Mass Index) eFigure. Correlation Matrix Among the Sphingolipid Species of Interest [file jamanetwopen-e2343854-s001.pdf]

Supplementary Online Content

Bockus LB, Jensen PN, Fretts AM, et al. Plasma ceramides and sphingomyelins and sudden cardiac death in the Cardiovascular Health Study. *JAMA Netw Open*. 2023;6(11):e2343854. doi:10.1001/jamanetworkopen.2023.43854

eTable 1. Mean Baseline Characteristics and Trends Across Quartiles of Sphingolipids Among 4612 CHS (Cardiovascular Health Study) Participants

eTable 2. Sensitivity Analyses for Risk of Sudden Cardiac Death per SD Higher Log Sphingolipid Level Based on 215 Events Among 4612 Participants

eTable 3. Mutual Adjustment of Species With Palmitic Acid, Cer-16 and SM-16, in Addition to Model 3 Adjustment

eTable 4. Interactions With Age, Sex, Race, and BMI (Body Mass Index)

eFigure. Correlation Matrix Among the Sphingolipid Species of Interest

This supplementary material has been provided by the authors to give readers additional information about their work.

© 2023 Bockus LB et al. *JAMA Network Open*.

eTable 1. Mean Baseline Characteristics and Trends Across Quartiles of Sphingolipids Among 4612 CHS (Cardiovascular Health Study) Participants

		Cer-16		Cer-20		Cer-22		Cer-24	
	**Mean (SD)**	Trend	Q1, Q4	Trend	Q1, Q4	Trend	Q1, Q4	Trend	Q1, Q4
**Age, years**	77 (5)		76, 77		77, 77		77, 76		77, 76
**Sex, % male**	41%		43, 38		48, 35		45, 39		40, 42
**Race, % black**	16%		18, 14		9, 21		14, 16		15, 15
**Education, years**	14 (5)		15, 14		15, 13		15, 13		14, 14
**Current smoker, %**	9%		7, 10		7, 11		7, 11		7, 11
**Current alcohol use, %**	28%		33, 22		35, 20		35, 19		26, 27
**Body mass index, kg/m²**	27 (5)		27, 26		26, 27		25, 27		26, 27
**HDL*, mg/dL**	53 (14)		55, 51		55, 51		57, 49		56, 50
**Triglycerides*, mg/dL**	144 (86)		120, 173		115, 176		109, 196		112, 19[illegible]
**LDL*, mg/dL**	128 (34)		115, 138		116, 137		111, 139		112, 14[illegible]
**C-reactive protein*, mg/L**	2.6 (1.2)		2.3, 3.4		2.0, 3.5		1.9, 3.3		2.1, 3.0
**BNP***	313 (712)		255, 395		326, 278		395, 235		383, 23[illegible]
**Troponin T***	9.0 (11.6)		7.8, 11.2		9.5, 9.0		9.3, 8.7		9.5, 8.8
**Heart rate**	64 (12)		63, 67		63, 67		63, 66		63, 66
**QT-interval**	418 (37)		422, 412		422, 413		424, 413		422, 41[illegible]
**QRS-interval**	94 (19)		94, 94		95, 94		95, 93		95, 93
**PR-interval**	174 (35)		176, 174		175, 170		177, 172		177, 17[illegible]
**LV Hypertrophy**	6%		5, 7		4, 8		5, 7		6, 7
**Systolic BP, mmHg**	135 (21)		134, 136		133, 136		132, 136		133, 13[illegible]
**Past smoker, %**	35 (56)		39, 28		41, 28		38, 33		34, 36
**Prevalent hypertension, %**	69%		69, 70		65, 73		64, 71		66, 71
**Lipid lowering therapy, %**	8%		11, 8		8, 10		9, 10		9, 9
**eGFR***	73 (19)		76, 69		73, 73		74, 72		73, 72
**Self-reported good health**	76%		79, 69		78, 71		76, 73		76, 74
**2 or more IADLs**	9%		7, 13		6, 12		8, 9		9, 8
**Prevalent COPD, %**	31%		27, 35		30, 33		30, 33		29, 33
**Prevalent diabetes, %**	13%		12, 16		11, 18		9, 21		10, 18
**Prevalent CVD, %**	30%		27, 34		27, 34		31, 30		31, 30
**Prevalent CHD, %**	24%		22, 27		23, 26		24, 24		24, 24
**Prevalent HF, %**	8%		7, 9		7, 9		10, 7		10, 7
**Prevalent Stroke, %**	7%		5, 10		5, 8		7, 7		7, 7
**Prevalent AF, %**	9%		8, 10		11, 9		13, 7		13, 6
**Prevalent MI, %**	11%		10, 12		11, 12		12, 12		12, 12

		SM-16		SM-20		SM-22		SM-24	
	**Mean (SD)**	Trend	Q1, Q4	Trend	Q1, Q4	Trend	Q1, Q4	Trend	Q1, Q4
**Age, years**	77 (5)		76, 77		78, 76		78, 75		78, 75
**Sex, % male**	41%		52, 31		57, 28		51, 29		44, 36
**Race, % black**	16%		8, 27		13, 20		11, 23		12, 22
**Education, years**	14 (5)		14, 13		14, 14		14, 13		14, 14
**Current smoker, %**	9%		8, 12		9, 10		8, 10		7, 11
**Current alcohol use, %**	28%		30, 24		28, 26		32, 21		25, 27
**Body mass index, kg/m²**	27 (5)		27, 26		26, 27		26, 27		27, 26
**HDL*, mg/dL**	53 (14)		48, 59		50, 57		51, 56		51, 56
**Triglycerides*, mg/dL**	144 (86)		162, 130		139, 147		134, 151		144, 145
**LDL*, mg/dL**	128 (34)		110, 143		107, 145		108, 145		111, 144
**C-reactive protein*, mg/L**	2.6 (1.2)		2.5, 2.6		2.3, 2.9		2.1, 3.2		2.4, 2.8
**BNP***	313 (712)		269, 361		479, 230		497, 214		440, 218
**Troponin T***	9.0 (11.6)		9.0, 9.2		11.8, 7.7		12.0, 7.4		10.9, 7.7
**Heart rate**	64 (12)		64, 66		64, 65		64, 65		64, 65
**QT-interval**	418 (37)		419, 415		420, 416		421, 416		420, 417
**QRS-interval**	94 (19)		95, 92		98, 92		97, 92		97, 92
**PR-interval**	174 (35)		178, 171		179, 171		180, 170		179, 172
**LV Hypertrophy**	6%		4, 7		5, 5		5, 6		6, 5
**Systolic BP, mmHg**	135 (21)		133, 137		134, 135		134, 136		134, 135
**Past smoker, %**	35 (56)		40, 29		36, 36		35, 34		31, 38
**Prevalent hypertension, %**	69%		68, 72		71, 68		69, 69		71, 68
**Lipid lowering therapy, %**	8%		12, 7		8, 8		9, 9		10, 8
**eGFR***	73 (19)		71, 73		69, 76		69, 76		69, 76
**Self-reported good health**	76%		78, 70		72, 77		72, 76		70, 78
**2 or more IADLs**	9%		6, 12		10, 7		11, 8		11, 6
**Prevalent COPD, %**	31%		29, 35		34, 31		32, 30		29, 31
**Prevalent diabetes, %**	13%		17, 10		15, 12		13, 14		14, 14
**Prevalent CVD, %**	30%		31, 30		37, 24		37, 26		36, 26
**Prevalent CHD, %**	24%		25, 23		29, 18		29, 20		28, 20
**Prevalent HF, %**	8%		9, 8		14, 5		13, 5		12, 5
**Prevalent Stroke, %**	7%		5, 9		8, 6		8, 6		8, 6
**Prevalent AF, %**	9%		10, 8		15, 5		15, 5		13, 4
**Prevalent MI, %**	11%		12, 11		16, 8		14, 9		14, 9

An expansion of Figure 1 to complete the data for all measured ceramide and sphingomyelin species. AF indicates atrial fibrillation; BP, blood pressure; BNP, N-terminal pro-B-type natriuretic peptide; Cer-16,

© 2023 Bockus LB et al. *JAMA Network Open*.

ceramide with acylated palmitic acid; Cer-20, ceramide with arachidic acid; Cer-22, ceramide with behenic acid; Cer-24, ceramide with lignoceric acid; CHD, coronary heart disease; COPD, chronic obstructive pulmonary disease; CRP, C-reactive protein; CVD, cardiovascular disease; HDL, high-density lipoprotein; HF, heart failure; IADLs, instrumental activities of daily living; LDL, low-density lipoprotein; LV, left ventricle; MI, myocardial infarction; Q1, quartile 1 (the mean or percentage of each characteristic among participants with a sphingolipid level in the lowest 25% of the distribution); Q4, quartile 4 (highest 25%); SM-16, sphingomyelin with palmitic acid; SM-20, sphingomyelin with arachidic acid; SM-22, sphingomyelin with behenic acid; and SM-24, sphingomyelin with lignoceric acid. The colored graphics show means or percentages of each characteristic across quartiles of each of the sphingolipids. Unadjusted linear and logistic regression models were used to assess statistically significant ($P < 0.0018$; 0.05/28 characteristics) associations of log-transformed sphingolipids with each characteristic; statistically significant positive trends are colored in blue, statistically significant negative trends are in red, grey indicates $P > 0.0018$.

© 2023 Bockus LB et al. *JAMA Network Open*.

eTable 2. Sensitivity Analyses for Risk of Sudden Cardiac Death per SD Higher Log Sphingolipid Level Based on 215 Events Among 4612 Participants

	Model 1				Model 2				Model 3			
	HR	95% CI		p	HR	95% CI		p	HR	95% CI		p
**Cer-16**	1.33	1.16	1.52	< 0.001	1.28	1.11	1.47	< 0.001	1.34	1.12	1.59	0.001
**Cer-20**	1.27	1.11	1.46	< 0.001	1.19	1.04	1.36	0.011	1.06	0.90	1.24	0.476
**Cer-22**	1.18	1.03	1.35	0.014	1.10	0.96	1.27	0.174	0.92	0.77	1.10	0.360
**Cer-24**	1.15	1.01	1.32	0.040	1.09	0.95	1.25	0.235	0.92	0.77	1.09	0.319
**SM-16**	1.17	1.01	1.35	0.032	1.25	1.05	1.48	0.012	1.37	1.12	1.67	0.002
**SM-20**	0.94	0.82	1.08	0.367	0.94	0.81	1.09	0.377	0.83	0.71	0.98	0.031
**SM-22**	1.00	0.87	1.15	0.996	0.98	0.85	1.15	0.839	0.84	0.70	1.00	0.054
**SM-24**	0.98	0.85	1.13	0.769	0.99	0.85	1.15	0.886	0.86	0.72	1.03	0.104

	Model 4				Model 5				Model 6			
	HR	95% CI		p	HR	95% CI		p	HR	95% CI		p
**Cer-16**	1.27	1.06	1.51	0.008	1.32	1.11	1.58	0.002	1.17	0.97	1.40	0.097
**Cer-20**	1.09	0.93	1.28	0.284	1.06	0.90	1.24	0.513	1.15	0.98	1.36	0.087
**Cer-22**	0.96	0.81	1.14	0.627	0.93	0.78	1.11	0.436	1.01	0.85	1.21	0.895
**Cer-24**	0.96	0.81	1.14	0.629	0.93	0.79	1.11	0.435	1.01	0.85	1.21	0.901
**SM-16**	1.30	1.07	1.58	0.009	1.35	1.10	1.65	0.004	1.19	0.98	1.46	0.083
**SM-20**	0.88	0.74	1.03	0.121	0.85	0.72	1.00	0.054	0.91	0.77	1.07	0.245
**SM-22**	0.89	0.75	1.06	0.181	0.85	0.71	1.02	0.079	0.93	0.78	1.11	0.425
**SM-24**	0.90	0.76	1.07	0.244	0.88	0.74	1.05	0.162	0.93	0.78	1.11	0.435

Hazard ratios and 95% CIs are presented.
Model 1 includes adjustment for age, sex, race, and study site.
Model 2 includes model 1 adjustment terms and additional adjustment for body mass index (BMI), treated hypertension, HDL, LDL, smoking, and prevalent diabetes and CHD.
In model 3, in addition to model 2 adjustment terms, ceramide with palmitic acid (Cer-16) and sphingomyelin with palmitic acid (SM-16) were adjusted for ceramide with behenic acid (Cer-22) and sphingomyelin with behenic acid (SM-22), respectively; ceramide with arachidic acid (Cer-20), behenic acid (Cer-22), and lignoceric acid (Cer-24) and sphingomyelin with arachidic acid (SM-20), behenic acid (SM-22), and lignoceric acid (SM-24) were adjusted for Cer-16 and SM-16, respectively.
Each of models 4, 5, and 6 includes additional adjustment terms that are additive to model 3. Model 4 includes prevalence of COPD, heart failure, atrial fibrillation, and chronic kidney disease (by eGFR).
Model 5 includes heart rate, QT interval, and QRS interval.
Model 6 includes CRP (C-reactive protein), NT-proBNP, and troponin-T.
Cer-16 indicates ceramide with acylated palmitic acid; Cer-20, ceramide with arachidic acid; Cer-22, ceramide with behenic acid; Cer-24, ceramide with lignoceric acid; SM-16, sphingomyelin with palmitic acid; SM-20, sphingomyelin with arachidic acid; SM-22, sphingomyelin with behenic acid; and SM-24, sphingomyelin with lignoceric acid.

© 2023 Bockus LB et al. *JAMA Network Open*.

eTable 3. Mutual Adjustment of Species With Palmitic Acid, Cer-16 and SM-16, in Addition to Model 3 Adjustment

	Alone (Primary Analysis Model 3)				Adjusted for the other -16 species			
	HR	95% CI		p	HR	95% CI		p
**Cer-16**	1.34	1.12	1.59	0.001	1.27	1.05	1.55	0.016
**SM-16**	1.37	1.12	1.67	0.002	1.23	1.00	1.52	0.053

Cer-16 indicates ceramide with acylated palmitic acid; SM-16, sphingomyelin with palmitic acid.

eTable 4. Interactions With Age, Sex, Race, and BMI (Body Mass Index)

	Age	Male	Black	BMI
**Cer-16**	0.63	0.05	0.16	0.36
**Cer-20**	0.76	0.22	0.64	0.77
**Cer-22**	0.80	0.10	0.24	0.36
**Cer-24**	0.85	0.14	0.03	0.80
**SM-16**	0.36	0.34	0.11	0.18
**SM-20**	0.98	0.58	0.61	0.26
**SM-22**	0.83	0.16	0.40	0.99
**SM-24**	0.90	0.48	0.25	0.87

P-values for interaction terms displayed.
Cer-16 indicates ceramide with acylated palmitic acid; Cer-20, ceramide with arachidic acid; Cer-22, ceramide with behenic acid; Cer-24, ceramide with lignoceric acid; SM-16, sphingomyelin with palmitic acid; SM-20, sphingomyelin with arachidic acid; SM-22, sphingomyelin with behenic acid; and SM-24, sphingomyelin with lignoceric acid.

© 2023 Bockus LB et al. *JAMA Network Open*.

eFigure. Correlation Matrix Among the Sphingolipid Species of Interest

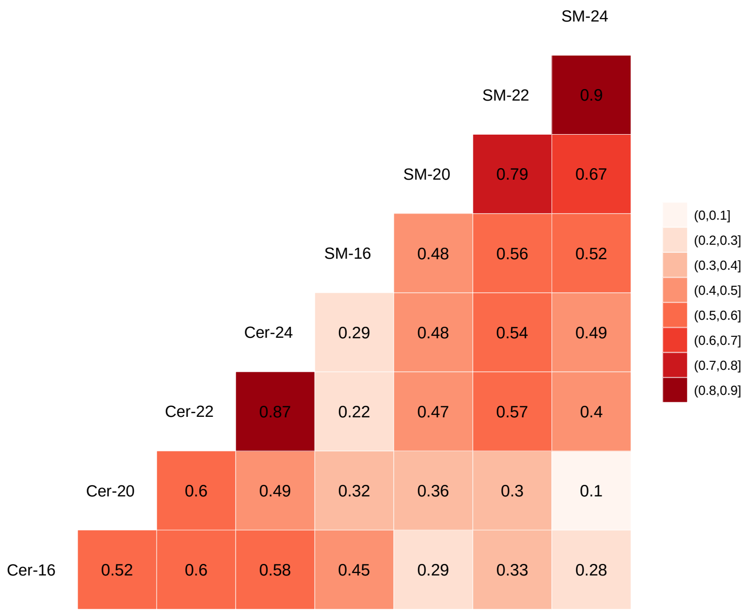

Cer-16 indicates ceramide with acylated palmitic acid; Cer-20, ceramide with arachidic acid; Cer-22, ceramide with behenic acid; Cer-24, ceramide with lignoceric acid; SM-16, sphingomyelin with palmitic acid; SM-20, sphingomyelin with arachidic acid; SM-22, sphingomyelin with behenic acid; and SM-24, sphingomyelin with lignoceric acid.

© 2023 Bockus LB et al. *JAMA Network Open*.
