# Supplementary material for: Plasma Ceramides and Sphingomyelins and Sudden Cardiac Death in the Cardiovascular Health Study
Source: JAMA Netw Open. 2023 Nov 17;6(11):e2343854. doi: 10.1001/jamanetworkopen.2023.43854 (PMC10656644; doi:10.1001/jamanetworkopen.2023.43854)
Supplement: Supplement 2. — Data Sharing Statement [file jamanetwopen-e2343854-s002.pdf]

Data Sharing Statement

Bockus. Plasma Ceramides and Sphingomyelins and Sudden Cardiac Death in the Cardiovascular Health Study. *JAMA Netw Open*. Published November 17, 2023. doi:10.1001/jamanetworkopen.2023.43854

Data
Data available: No

Additional Information
Explanation for why data not available: The authors are not authorized to share data from the Cardiovascular Health Study (CHS). Information to request the use of CHS data can be found at https://chs-nhlbi.org/.
